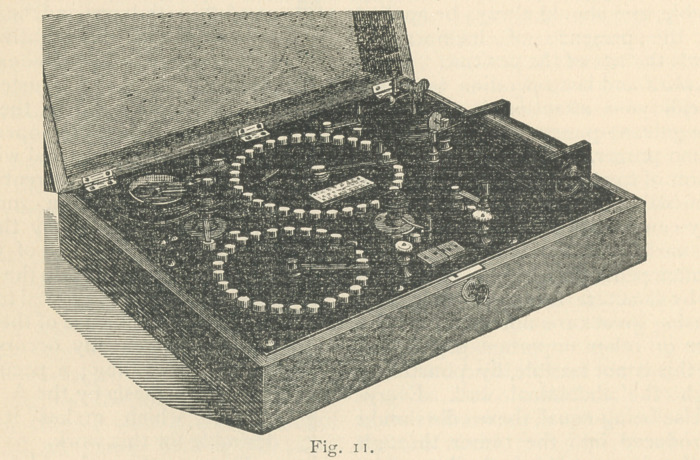# The Value of the Galvanic Current in Gynæcology

**Published:** 1887-07

**Authors:** Franklin H. Martin

**Affiliations:** Chicago, Ill., Professor of Gynæcology in Chicago Policlinic


### THE VALUE OF THE GALVANIC CURRENT IN GYNÆCOLOGY.

BY FRANKLIN H. MARTIN M. D., CHICAGO, ILL., PROFESSOR OF GYNÆCOLOGY IN CHICAGO POLICLINIC.

Electricity, just at present, is receiving that merited attention from the medical profession that all therapeutic measures of great value are in time bound to command. The time is past when a physician of rank can afford to ignore the power for good that is stored away in a well-regulated electric battery. Neither can a physician any longer, who values his reputation for intelligence, be content to give " electrical treatment " in the haphazard manner that has been prevalent among the majority of men until within the last few years.

Electricity has become a known quantity; it can be generated, measured and stored at will. The amount of work that can be *accomplished* by a given current can be figured to a mathematical certainty. It is governed by certain inexorable laws which make it a powerful agent for good or harm according to the intelligence of the one to whose keeping it is intrusted.

In this article I wish to give a cursory glance at what can be accomplished in the treatment of certain gynæcological diseases by the bipolar application of a continuous galvanic current of electricity. I mean by the bipolar method, the use of two electrodes, one from each pole of the generator, arraigned upon the body in such a manner that the diseased portion lies in the line of and constitutes a portion of the circuit.

The following effects are utilized in the bipolar method of applying galvanism; 1st, the local effects of the electrodes; 2nd, the electrolytic effect of the current; 3d, cataphoric action of the current; 4th, anæsthetic effect of the current.

The local effect of the electrodes vary with the character of electrode, the particular pole employed and the density and strength of the current. In gynæcological practice one electrode only at a time, as a rule, is considered the active. As this active electrode is ordinarily placed in the vagina or uterus, it is also called the internal electrode. This internal active electrode may be either positive or negative, according to indications, and should be composed of conducting metal, either covered with moist sponge, cotton or chamois, or uncovered, as indications (which will be referred to hereafter in detail) may demand. The external electrode, which is usually passive, is for the purpose of closing the circuit in such a manner that the current in passing between it and the internal electrode will traverse the diseased tissue, and is placed upon the integument. This electrode is constructed of material that makes it valuable as a conductor, while it diffuses the current sufficiently to avoid pain. It may be an ordinary flat sponge electrode (Figs. 1 a and b), or a membranous electrode on the principle of the one represented in Fig. 8.

Fig. 1 a.

Fig. 1 b. Small flat sponge electrode.

The first effect locally on living tissues in which the active positive pole comes in contact when a current is gradually increased in strength, is slight irritation accompanied with redness. As the current is increased the surface becomes contracted and a sharp, smarting sensation is experienced; when carried still further the surface becomes contracted and hard, and coagulation of the deeper tissues around the electrode takes place; if the process is carried still further, a deep, hard eschar surrounding the electrode is the result. The effect produced here is identical with that obtained by a caustic acid. It is produced by the acid radicals of the decomposed tissues rallying around the positive pole. This pole is therefore called the acid pole.

The first effect locally on living tissue with which the active *negative* pole electrode comes in contact when the current is first rendered perceptible, is an appearance of moisture appearing on the surface of the electrode; as the current is increased the moisture increases, and a slight bubbling of gas is noticed from around the electrode; as the current is further strengthened a white eschar is found beneath the electrode, and as the current is still further increased a deep white slough is produced with an abundant flow of liquid and escape of gas.

The effect produced at this pole is very similar to that caused by caustic alkali. The negative pole is called the alkaline pole. To be able to utilize all the effects of the poles described above, a means of generating electricity of a working strength of from 5 to 500 milliampères should be at hand. In order to obtain uniform results and to guard against accidents in manipulating strong currents a galvanometer should always be used. See Fig. 2.

Fig. 2.

*Electrolysis*, the principal action of the galvanic current that we seek in causing the absorption of pathological tissues, is not a new phenomenon in electro-chemistry. It is the power that a galvanic current has of resolving certain compound bodies into their constituent elements. By this action the more loosely combined elements of certain compound molecules of any pathological tissue that the current may be traversing are relaxed and their deposit accomplished where they will be carried from the system.

*The Cataphoric Action* of the galvanic current should also be recognized as playing its part in the promotion of absorption. It is by this property that fluids are determined in mass from the positive to the negative pole. This action, in producing an unnatural turgescence of fluid at the negative pole, favors its osmosis into the surrounding absorbents, and thereby accomplishes its removal.

*The Anæsthetic Effect* of the galvanic current is of great value in gynæcological work. In some as yet inexplicable manner galvanism has a power of relieving pain, especially that variety of pain included under the ordinary term neuralgia.

The pathological conditions which I wish to discuss and to utilize the above points in, and in which I have received marked beneficial results and not a few cures in gynæcological practice, will be included under the following headings:

1. Chronic Cellulitis and Peritonitis.
2. Chronic Ovaritis and Salpingitis.
3. Chronic Subinvolution of Uterus.
4. Chronic Hyperplasia Uteri.
5. Uterine Stenosis.
6. Laceration of Cervix Uteri.
7. Peri-Uterine Hæmatocele.
8. Uterine Fibroids.

*Chronic Cellulitis and Peritonitis.*—Under this head I wish to consider all conditions in which inflammatory ex-udates have been deposited in the fe-male pelvis anywhere, caused by a general or local peritonitis, perimetritis, parametritis, or inflammation of the cellular tissue surrounding the uterus and vagina, or in the broad ligaments. Under this head we meet with some of the most aggravating difficulties that the gynæcologist is called upon to treat. Among the objective symptoms we fiind: Hardened, thickened condition of the anterior or posterior wall of the vagina; thickened deposits in Douglas' cul-de-sac, limiting the movements of the cervix; thickened masses between the anterior portion of the cervix and the bladder, with adhesions; thick inflammatory masses laterally between the folds of the broad ligaments, displacing the uterus and interfering with its motion; and in many cases we have a general thickening on all sides, with an immovable womb and tenderness in every direction. The subjective symptoms accompanying these various conditions are as varied as the objective. Besides the many local symptoms, we may have any or all of the organs of the body controlled by the sympathetic nervous system, involved in a sympathetic distress. The local symptoms include all forms of distress that may arise from interference with the functions of the pelvic organs. Constipation, sacralgia, dysuria, dysmenorrhœa, together with acute neuralgias in regions where twigs of nerves and sensitive nerve-ganglia are imprisoned in the inflammatory exudates.

While one or more of these multiform conditions are met with almost every day, we are almost helpless in our efforts to remove these inflammatory deposits and thickenings after they have become organized. True, we can promote absorption slowly, by the use of hot water, iodine, glycerin, massage, etc., and in cases of small deposits we may get absolute cures. It is in just these cases that electrolysis is indicated. Electrolysis, if properly manged, will promote the absorption of these exudates very rapidly, and in my hands has been the means of relieving a great many very severe symptoms together with their cause. I have a number of cases which properly come under this head, that I have treated with beneficial results, in not only relieving the symptoms for which I was consulted, but in which I have also removed the inflammatory deposits.

It will be impossible, however, in this one short article to do more than give the description of my method in each variety of cases. In these cases the treatment is applied in such a manner as to include as much of the inflammatory matter in the electric circuit as possible at each *séance*, and is varied so as to include all in the course of two applications. The external electrode is connected with the positive pole of the battery. This electrode should be a flat sponge hand-electrode, similar to the one manufactured especially for me by the McIntosh Galvanic Battery Co., of Chicago (Fig. 1 b). It is a flat metal, the length of the hand, and about two and a half inches wide, covered on its inner surface with sponge, while the outer surface is covered with rubber, over which a wide band is fastened at either end, through which the hand is passed. This electrode is worn on the hand of the operator, and is narrow enough not to interfere with the flexion of the fingers. By means of this the operator can locate the uterus as in bimanual manipulation, and control the direction of the current perfectly. The negative electrodes employed are of two forms and are used on alternating days. One an ordinary rectal electrode of metal insulated with hard rubber to within one inch and a half of the end, and about one-fourth of an inch in diameter, with an olive point (Fig. 3); the other an ordinary vaginal electrode of metal, insulated to within an

Fig. 3. Fig. 4.

inch of the extremity, with the extremity round and about three-fourths of an inch in diameter (Fig. 4). When the object is to promote absorption of pathological tissue some distance removed from the negative pole, it is well to have the metal covered with a moist piece of chamois.

The vaginal electrode is introduced into the vault of the vagina and connected by means of flexible wires with the negative pole of the battery. The hand electrode is controlled by the right hand, as the current is gradually increased a cell at a time by the left hand. The right hand with the sponge electrode, well moistened, is pressed firmly down over the uterus until the patient feels a smart, burning sensation of the skin. Then the vaginal electrode is pressed firmly into the vaginal vault and kept in one position throughout the *séance*. In these positions the electrodes remain and the current is allowed to act for ten minutes. Each alternate day the rectal electrode is used, the metal portion pressed firmly against the thickened anterior wall in such a manner as to cause the current to pass through the hardened exudate. Thus, in the course of a few settings, all portions of the pelvic cavity affected, help to complete the circuit of the battery, and obtain the effect of the electrolytic action.

*Chronic Ovaritis and Salpingitis* are so intimately connected with all forms of pelvic inflammation that they may almost properly be considered under the head of chronic peritonitis and cellulitis of the pelvis. Ovaritis and salpingitis are so inseparable in their inflammatory diseases that in the late fashionable craze to unsex the women they have both invariably disappeared together.

In conditions of salpingitis and ovaritis, with inflammatory deposits, whatever the cause is, I am convinced great relief can be obtained, as far as the removal of the deposits will give relief, by the judicious employment of electrolysis. I am certain that in a number of painful and serious conditions of this kind I have obtained relief. At any rate, the method is well worth trying before advising the removal of the ovaries. There is very little relief to be obtained by any other non-surgical procedure.

The following case is a well-marked one of the kind, and one in which I think a cure was effected:

A young married woman, twenty-six years of age, with no children, consulted me for a pain in her side, which had been gradually increasing in severity for three years. The pain was constant, with excessive exacerbations for two days before each menstrual period, at which time there was also pain, though not so intense, on the right side. There was tenderness on pressure in the left ovarian region, and if the pressure was persisted in it increased the pain so that it resembled that experienced before menstruation. In the left broad ligament a little body was discoverable which seemed like a prolapsed ovary. It was considerably enlarged and perfectly immovable. The right side was free from tenderness, and contained nothing abnormal. The pain of that side was evidently of a sympathetic nature.

For this patient electrolysis was recommend three times a week. The current that could be borne at the first few sittings was very weak, three or four milliampéres being sufficient to cause increased pain in the parts. The vaginal electrode (the negative) was crowded well up on the left side of the vaginal junction, and the external sponge electrode passed gently over the region externally, so as to include the tender ovary and tube in the circuit. The patient soon became more tolerant to the current, and it was increased within two weeks to as many as fourteen milliampères. The patient got relief from the first application. The treatment was contined for about nine weeks. After the second week the continuous pain had entirely disappeared, although she suffered some at the first menstrual period. At seven weeks the pain and tenderness had disappeared; her menstruation was free from pain. Upon manipulation the ovary was found much reduced in size, movable, and free from all abnormal tenderness. Treatment was suspended after nine weeks.

*Chronic Subinvolution.*—This condition is so frequently met with that I have had numerous opportunities of witnessing the beneficial action of galvanism in its treatment. I have noticed it gradually recduce in size while treating other pelvic conditions with galvanism where it was necessarily brought into the circuit.

The internal electrode used here is a cervical electrode of metal, cup-shaped and insulated except the concavity of the cup (fig. 5). This is introduced in such a manner as to have the cup support the cervix uteri. The circuit is closed by applying the hand sponge electrode over the fundus. A current from ten to twelve milliampères is tolerated without harm, each *séance* lasting ten minutes.

Fig. 5.

*Hyperplasia of the Uterus.* — This condition, which in many respects resembles subinvolution, can properly be considered now. It does not necessarily, although usually, occur in women who have borne children. It may be simply an inflammatory condition of the connective and fibrous tissue in a chronic, subinvoluted uterus, or it may be a general hyperplasia of the uterus, caused by the irritation of a lacerated perineum or cervix; or it may be caused from the irritation of repeated menstrual congestions, which is made abnormal by a flexion or displacement of the uterus; or an error of nutrition from a general nervous derangement may be the exciting cause. *Whatever the causes are*, we find a large, tender uterus, a thickened, catarrhal condition of the endometrium, oftentimes an inflamed condition of the cervical glands, with the thick, albuminous discharge welling out, and very often a well-marked abdominal displacement from the sheer weight of the organ. The condition is one of hypertrophy of the normal constituents of the organ from congestion and inflammation. I have several times seen this condition yield to the chemical effect of the galvanic current when using it in other connections. In a condition often accompanying this I have seen very pronounced results, and it is worthy of mention because of the unsatisfactory results by the ordinary methods of treatment of the difficulty. I refer to *inflammation of the cervical glands*. This disease, with its characteristic tenacious albuminous discharge, every physician has more or less to do with. The ordinary forms of treatment, where cure is accomplished are harsh, and unsatisfactory in the extreme. Destruction of the deep gland tissue by curetting, cauterizing or cutting are the only methods that have heretofore given satisfactory results. These glands can be destroyed, or their condition of chronic inflammation reduced by the effect of repeated applications of a metal bulb of the negative pole of a galvanic battery. I first noticed this condition yield while treating other forms of pelvic trouble with electricity, where it was desirable to pass a metal electrode into the cervical canal. One-half dozen thorough applications of this description, using a bulb electrode similar to those used for stricture of the urethra (Fig. 6), and gradually increasing the size of the bulb, will, I think, cure the most obstinate cervical catarrh. A current of from fourteen to twenty milliampères can be used without producing any pain. I pass the bulb of the electrode through the internal os at each application.

*Strictures of the Uterine Canal* I have removed by electrolysis in a large number of cases. It was in this condition, at the suggestion of Prof. De Laskie Miller, of Chicago, that I first used electrolysis. That gentleman had been using it with success for some time before my first attempt. It matters not what the condition is that produces the stenosis, or in what portion of the canal it exists. It has all the advantages over gradual dilatation, divulsion or cutting, that electrolysis has in the treatment of male urethral strictures over gradual dilatation, divulsion or cutting, viz.: painlessness, permanency and, in cases of gradual dilatation, promptness.

The method of operating on these strictures is very similar to that employed in strictures of the urethra. A small staff of soft metal covered with hard rubber, upon the end of which one of the graduated set of olive-shaped metal bulbs may be screwed, is used for the uterine electrodes. After making out the position of the uterus by manipulation, or by passing a small, flexible metal probe, the staff of the electrode is made to conform with the supposed shape of the canal (Fig. 6). After this a flat sponge-electrode, attached to the positive plate of the battery, is applied to the thigh or abdomen of the patient, the internal electrode is passed into the cervix, either through a Sims speculum or a wide bivalve, and while the cervix is steadied with a strong tenaculum with one hand, the electrode is guided by the other into the cervix until it finds its first obstruction, the current is gradually increased, while very gentle pressure is made on the electrode until it passes in the course of the canal all the obstructions. Usually the smallest canal can be passed at the first sitting of a bulb the size of a No. 7 or No. 8 English catheter. This can be repeated with larger bulbs, if desirable, for three or four times, when it will be found that the canal is permanently enlarged.

Fig. 6.

*Laceration of the Cervix Uteri.*—This is a condition which, of course, cannot be repaired by electrolysis. I hope it will not be considered too enthusiastic, however, if I assert that temporary relief can be obtained from the results of laceration of the cervix by the proper use of electrolysis. The painful consequences following this condition of laceration are due in great part to the formation ot cicatricial tissue in the fissure, to degeneration of the mucous follicles, to obstructed circulation in the neck, and to connective tissue changes and a congested hypertrophy.

By experience in just this condition, when the privilege of trachelorrhaphy was withheld, or when from some other good reason I was deterred from operating, I have obtained very gratifying *temporary* results. I emphasize *temporary*, because time enough has not passed since the operations to warrant me in saying permanent. The cervical cup (Fig. 5) is used for the internal electrode in the treatment of these cases, with an occasional application to the canal of the cervical bulb electrode (Fig. 6).

This method of treatment is so prompt in relieving this condition that it is destined to become very popular as a temporary measure, or as a general measure when there are insurmountable barriers to an operation. As a preparatory measure for trachelorrhaphy it surely possesses very superior claims.

*Peri-Uterine Hæmatocele.*—Dr. Apostoli recently described a method of treating this condition by means of negative-galvano-puncture, as follows: "The chemical caustic action of the continuous current is utilized in making an opening into the tumors. The opening thus made is, in character, a non-retractile fistula, with tendency to remain open, and with adhesions between the pathological cavity and the external mucous membrane. * * * * The advantages of this method are that, on account of the adhesions formed, the danger of opening is lessened, and the cicatrix left by the negative eschar is slight and non-contractile. A further after-effect of this method of utilizing the chemical caustic action of this current is that the nutrition of these pathological cavities is modified, leading to retrograde metamorphosis."

*Fibroid Tumors of the Uterus.*—While there are many methods described for the treatment of this troublesome malady by electricity, I shall content myself in this paper to a description of a method that I have pursued for some time with very gratifying success. The method that I refer to is a slight modification of that devised and practiced by Dr. Apostoli, of Paris.

*Consideration of Tumors.*—I will not go into a long discussion of the history, causes and pathology of fibroid tumors of the uterus, because it does not particularly bear upon the subject before us. However, to facilitate a description of my method of treatment, I will make the old division according to location into: 1, submucous; 2, interstitial; 3, subperitoneal. The nomenclature of this division sufficiently explains itself. To still further expedite matters, allow me to divide these tumros according to their condition into the hæmorrhagic and the non-hæmorrhagic. By hæmorragic we will include those tumors that produce from a simple excess of the menstrual flow to the most alarming continuous hæmorrhage. The non-hæmorrhagic will include all others.

In the treatment of these tumors we utilize all the effects of the galvanic current heretofore enlarged upon, viz.: 1, the local effects of the electroyis; 2, the electrolytic effect of the current; 3, cataphoric action of the current; 4, anæsthetic effect of the current. With the exception of the first, the local effect of the electrolis, it will not be necessary for me to dwell upon these points. I wish, however, more specifically to speak upon this point in this connection.

The effect of the positive pole is termed by Dr. Apostoli the galvano-caustique effect of the positive pole. This is an action of a great deal of importance, and from which a great advance is contributed in the treatment of hæmorrhagic fibroid tumors of the uterus. The phenomenon is obtained only by the employment of a very strong current, from 50 to 1,000 milliampère strength, and concentration of this current at the point of contact of the positive pole of the battery to the tissues. This electrode must be of small size, and of some unattackable metal. The effect obtained upon vascular tissues or mucous membrane by thus concentrating the current is to produce an eschar. This eschar, however, if the current has been proper, will be found to be simply a coagulation and a hardening of the mucous membrane and the tissues beneath it for some little distance. This process of contraction and coagulation modifies the calibre of the vessels of the circulation so that hæmorrhages are less liable to occur at the point of application; at the same time it does not destroy the circulation sufficiently to produce strangulation and death of the part. There is nothing that will so effectually stop all forms of excessive hæmorrhages or leucorrhœa, without producing a troublesome slough and a subsequent contraction, as this particular application of electricity when made to the mucous membrane of the uterus.

The local effect of the negative pole is the opposite of that of the positive when the current is strong and concentrated. The action of this pole is one of liquefaction instead of hardening and coagulation. Its eschar resembles much that of a caustic alkali.

*The Electrodes* used in the treatment of fibroid tumors by Dr. Apostoli's method are few in number. Two electrodes are always necessary at every operation. One of these is applied externally on the surface of the body, the other in some form internally. Where powerful currents are employed, such as I have described, the first object is to devise means of conducting them through the parts desired, without producing harm to innocent tissues, or pain to the patient. One pole, the internal, is usually the active one. This is either a sound, that fits accurately the uterine canal, or a pointed electrode which enters a presenting portion of the tumor. This is constructed of unattackable metal, platinum when in form of sound, platinum and iridium when in form of needle. Some means of insulating the vaginal portion of the electrodes must be devised. (Fig. 7.) Needles used in electrolysis should be insulated up to within one inch of the point in order to protect external tissues. After the proper internal electrode has been selected and placed in position, and before the current is turned on, the circuit of the battery must be closed by applying some form of convenient electrode externally that will give the minimum resistance without excessive pain. This has been one of the most difficult things to accomplish in the use of strong currents. This external electrode should be placed upon the abdomen in as close proximity to the internal one as possible. It should have a large surface in order to diffuse the current. All parts of the surface should conduct equally, and fit accurately into all irregularities of the surface. Dr. McIntosh has succeeded in constructing for me this electrode (Fig. 8). A concave disk of soft metal, of appropriate dimensions, has loosely stretched over its concavity an animal mem-membrane which is fastened to its circumference securely enough to render it watertight. Between the concavity of the disk and the membranes is left a space one and one-half inches in thickness, which is filled with hot water or a saturated solution of chloride of sodium. The electrode is filled through a stopper on the surface of the metal; the connections are also made from this surface.

Fig. 7.

Fig. 8.

This electrode, when filled, is applied to the surface of the body so that its membrane surface is in contact with the skin. It adapts itself accurately to all irregularities, covers a large surface, and causes a diffusion of the current so perfectly, and makes connections so complete, that I have been able repeatedly to use a current of from 200 to 1,000 milliampères, without the necessity of an anæsthetic.

*Details of Application.*—There are three distinct operations that are called for in the rational treatment of fibroid tumors of the uterus by electrolysis. They vary according to symptoms and conditions. The first that I will consider is for the relief of excessive hæmorrhage. I consider this of first importance because it is one of the most distressing symptoms we meet with in dealing with these difficulties, and a symptom which often baffles every other resource.

I will not go into a description of the different conditions that *may* be present in hæmorrhagic fibroids, but will confine myself to the few conditions that must be present to allow of benefit from this treatment. We have excessive hæmorrhage, which may be continuous or periodic. The hæmorrhage is from the cavity of the uterus. The uterine canal must be accessible to a flexible probe. There must be no acute metritis, peri- or para- metritis present. With these requisites, positive and negative, we may proceed to operate. An assistant, if the patient is reasonably strong, is not necessary. The application is preferably given in the office.

The patient's clothing is loosened, and she is instructed to assume the dorsal position upon an operating chair or table. A speculum is introduced or not, according to convenience of operator, and the direction, size and depth of the uterine canal is ascertained by means of a soft metal probe. A uterine sound electrode composed of pure platinum (Fig. 7), corresponding as nearly as possible with the size of the uterine canal, is selected, and made to conform to the general direction of the canal, as indicated by the probe. This electrode is then introduced to the bottom of the uterus, and the insulating shield is pushed up until it touches the cervix and covers perfectly the intra-vaginal portion of the metal. When the electrode has been satisfactorily applied and connected with the positive pole of the battery, Martin's large abdominal electrode (Fig. 8), properly prepared and attached to the negative pole of the battery, is applied to the lower portion of the patient's abdomen in such a manner as to bring its whole surface in contact with the skin.

After the electrodes have been securely placed and the connections are found to be perfect, the operator should commence turning on the current. This should be done very gradually at first, in order that the patient may experience no shock. If the patient complains of a dull pain internally while the current is being increased, the operator should stop for a few seconds, and he will often find that the pain will cease, after which the current can again be increased to the desired strength without excessive pain. At the first not more than a 50-milliampère current should be used. If the patient bear this well, it should be increased at each succeeding operation until a strength of from 100 to 500 milliampères is obtained. The first operation should last about five minutes, and if well borne the succeeding ones can safely be lengthened to ten or twelve minutes. In finishing the operation the current should be decreased in strength a cell at a time, very slowly, until it is entirely excluded. After the operation the patients should remain quiet for half an hour, when they can return to their homes, with instructions to keep very quiet for at least twenty-four hours.

This operation has two effects. It checks hæmorrhages, and reduces the size of the tumor. The local coagulating effect of the platinum electrode upon the inner surface of the uterine canal checks the hæmorrhage, and the electrolytic effect of the powerful current through the tumor favors its absorption. A number of repetitions of the operation are necessary to control severe hæmorrhages. It is impossible, at one sitting, for the internal electrode to come in contact with all the surface of the canal, no matter how much pains may be taken to make an accurate adjustment. Nothing but repeated operations can accomplish this. Unless the current is too strong there will be but very little subsequent sloughing at the place of contact of the positive pole. If troublesome sloughing *should* occur after an application, a somewhat weaker current should be used afterwards. It must be remembered that coagulation, and not cauterization of the tissues is the point sought.

The second operation to which I will call your attention is for the reduction of tumors that have grown in such a manner that they have distended or occluded the uterine canal so that it will not admit a sound electrode, and thereby renders intrauterine *treatment proper* impossible. In these cases an *artificial* canal should be established in the obstructing portion of the tumor by means of negative galvano-puncture entering the tumor from the cervical canal.

For this operation the patient is also placed in the dorsal position. The position of the cervix is ascertained, and by aid of a proper speculum a sharpened probe of platinum and iridium is thrust a safe distance into the center of the presenting fibroid. The vaginal portion of the electrode is properly insulated as in the other operation, and is then connected with the *negative* pole of the battery. The abdominal electrode is attached to the positive pole and applied as for the previous operation, with proper precautions the current is gradually turned on until the desired strength is obtained, this being as high as 150-milliampères, often without pain or harm to the patient. Contrary to ordinary expectations, the *pain* produced by this operation is not sufficiently severe to require an anæsthetic, and with the exception of the first *séance*, when the new canal is established, the succeeding treatment is no more disagreeable than the ordinary use of the internal electrode in the uterine canal. Before any internal needle or puncture operation a vaginal injection of 1 to 5,000 bichloride of mercury should be given, and repeated each day as long as the treatment is continued. An internal of about five days should elapse between the first and second application, and the duration of the *séance* should be from five to ten minutes.

The first effect derived from the employment of this method is the establishment of a new channel to take the place of the distored and obstructed uterine canal and by which subsequently the tumor will be treated. The second effect is the direct electrolytic action of the current upon the growth. The channel left after the withdrawal of the probe is somewhat larger in diameter than the electrode itself and will remain for a number of days penetrable to the probe. There is but slight suppuration from its surface, but should there be any considerable hæmorrhage from the artificial canal, one application of the electrode with the current reversed, making the positive pole the internal, will give relief. One imperative point in these operations is the proper selection of poles to be employed. The immediate effect of the negative pole is to liquefy the tissues with which it comes in contact, like a caustic alkali, and it should be selected for the establishment of a canal, while the effect of the postive pole is to coagulate and harden tissues coming in contact with it, like a caustic acid, and it should always be employed to check hæmorrhage either from the artificial canal or from the natural uterine canal itself. Therefore, for the rapid reduction of a tumor the negative pole is decidedly preferable, and should always be applied unless the presence of hæmorrhage demands the use of the positive.

*The third* and last operation to which I will call your attention is the extra-uterine galvano-puncture, or the needle operation proper. Tumors that call for this form of treatment are those that are not amenable to the other two forms already considered.

This variety, usually of a subperitoneal type, often pedunculated, can be reached either by puncture through the vaginal wall, using great care not to injure the bladder or other important organs, or, where this is not feasible, by puncturing through the abdominal wall. Everything else being equal, the needle should be introduced into the tumor through the vagina, because if it is at all possible, the tumor lies in contact with the vagina; therefore the tissues that it is necessary for the needle to traverse are unimportant, while the abdominal wall with its fat, muscle, and peritoneal layers is too formidable to penetrate even with a needle unless actuated by absolute necessity. If the puncture is to be made through the vagina, a thorough vaginal injection of the bichloride solution should be employed, and for this operation, unless the patient has more than ordinary fortitude, an anæsthetic should be administered. For this purpose I employ, as preferable, the mixture of equal parts of alcohol, ether and chloroform, on account of its prompt action and transient effects.

After it has been definitely determined that this operation is the only alternative the patient is placed in the dorsal position, and all instruments being in readiness, the anæsthetic is pushed promptly to a surgical degree and then entirely withdrawn. A suitable speculum is introduced, and a needle with a proper curve and insulated with hard rubber to within one inch of the point is thrust through the vaginal wall into the tumor. (Fig. 9.). The position of the bladder should have been ascertained by means of a probe or catheter, before commencing work. When the needle has been satisfactorily adjusted the speculum is carefully withdrawn and the needle connected with the negative pole of the battery. The same external electrode should be adjusted with the same care, and in the same manner as in the other operations described, and connected with the positive pole. The current is now gradually turned on until a strength as indicated by the galvanometer is reached of 500 to 1,000 milliampéres. In the meantime the patient is allowed to come out from the influence of the anæsthetic, which usually occurs without any struggling; a peculiarity of the anœsthesia by the A. C. E. mixture, which makes it very desirable for this work.

Fig. 9.

This operation should last for about eight minutes the first time, and subsequently, if it is deemed desirable, a longer time can be given. When the operation is terminated, and the current has been gradually withdrawn, the needle will be found loosened, and its removal is accomplished with ease and very little pain.

The abdominal operation is performed where the tumor is of the large subperitoneal variety, that cannot be safely attacked from the uterus or vagina, and where the tumor rises above the cavity of the pelvis and is in contact with the abdominal wall. Here, after necessary antiseptic precautions have been carried out, the needle is thrust through the abdominal wall into the substance of the tumor at its most prominent part, and is then connected with the negative pole of the battery. The other electrode, the same variety as described in the other operation, should be placed upon the abdomen in close proximity to the needle. The current should be 300 to 1,000 milliampères strength, and the duration of the operation ten minutes.

Fig. 10.

Fig. 11.

*Battery.*—The current of electricity employed in gynæcological work should be uniform and without interruptions. Any means of generating electricity which will be practicable and at the same time answer the above requirements will be suitable for our purpose. I know of nothing better than some form of the chemical battery. For the ordinary purposes, where a working current required is not more than 50 to 75 milliampères, some form of the ordinary zinc and carbon cell with a solution of dilute sulphuric acid and bichromate of potash is suitable. The battery that I employ for this work is the one manufactured by McIntosh & Co., of Chicago. (Fig. 10.) It combines the advantages of many others, is simple in construction, easily cleaned and recharged, and combines a good faradic current with the galvanic. This battery is portable. For accomplishing more decided work and for a stationary battery in the office, the gravity cell, the diamond carbon, or the Lés Clàuchi cells are recommended. I use the ordinary crow-foot gravity cell. These should be connected with a switch-board, arranged in a manner that any portion of the battery can be used at will. For office battery I use 115 gravity cells, which are stored in a convenient closet and connected with a McIntosh switch-board (Fig. 11) by means of a cable of wires.

## Figures and Tables

**Fig. 1 a. f1:**
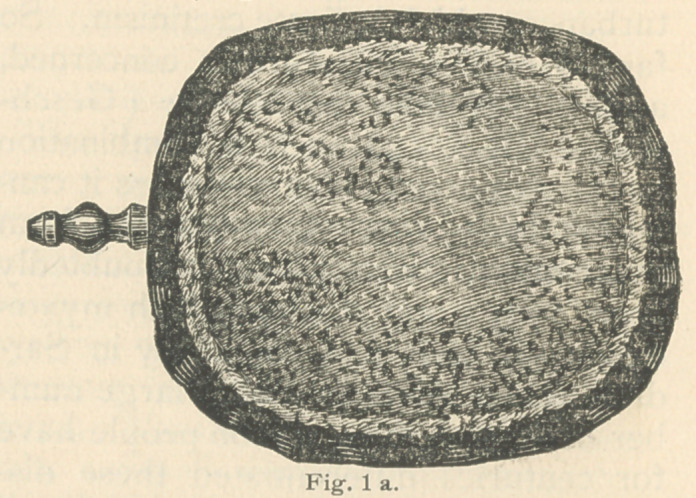


**Fig. 1 b. f2:**
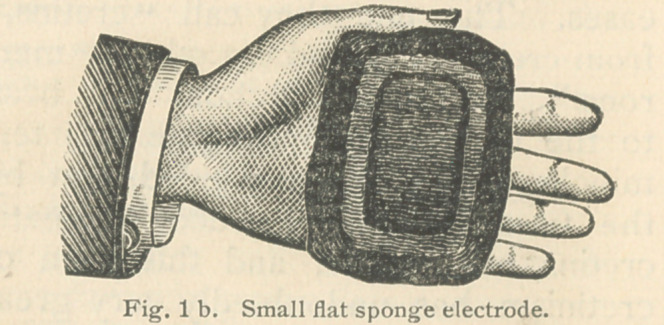


**Fig. 2. f3:**
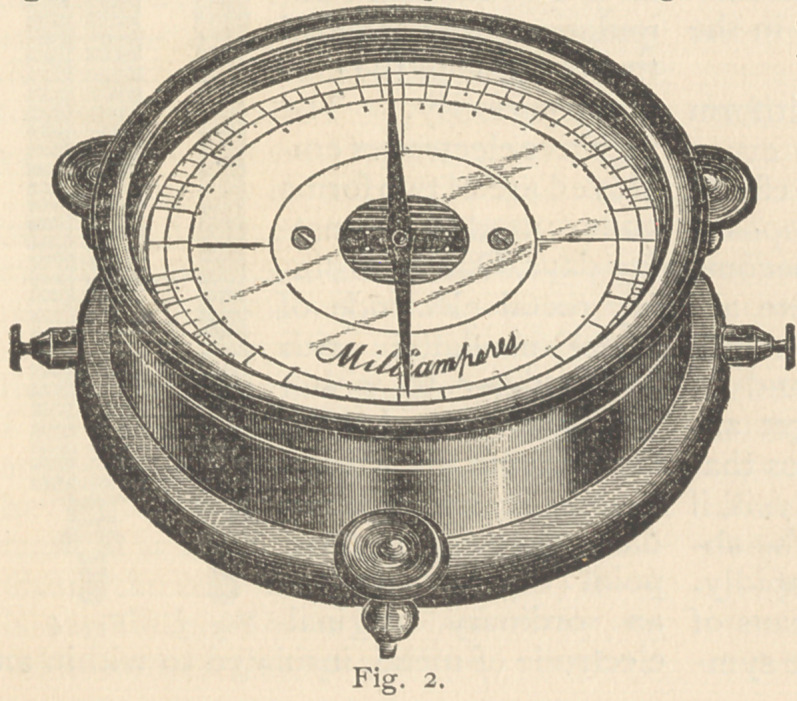


**Fig. 3. f4:**
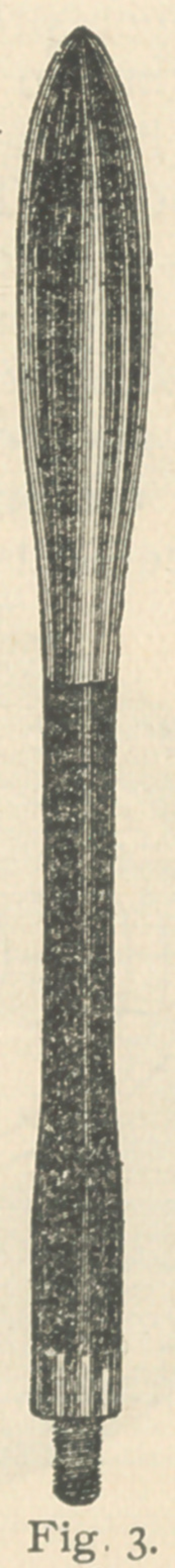


**Fig. 4. f5:**
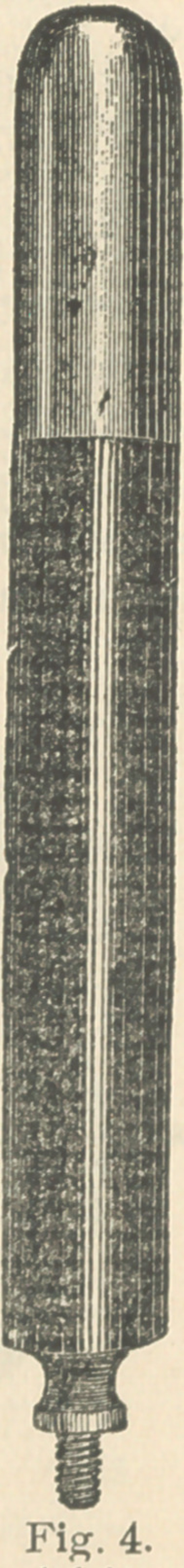


**Fig. 5. f6:**
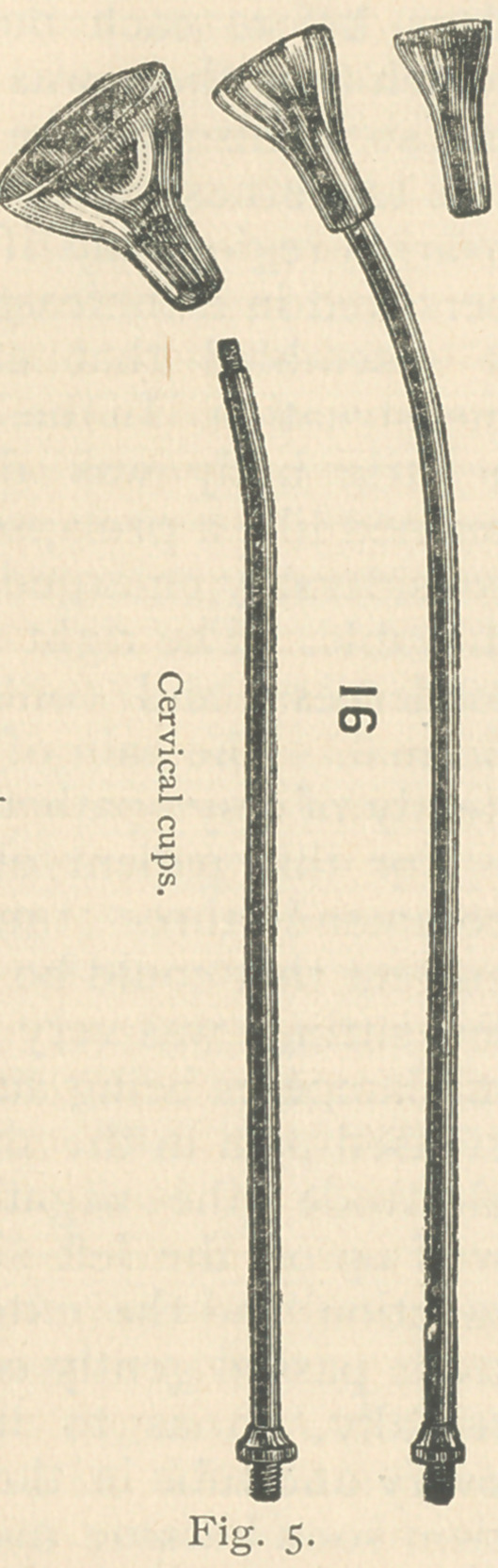


**Fig. 6. f7:**
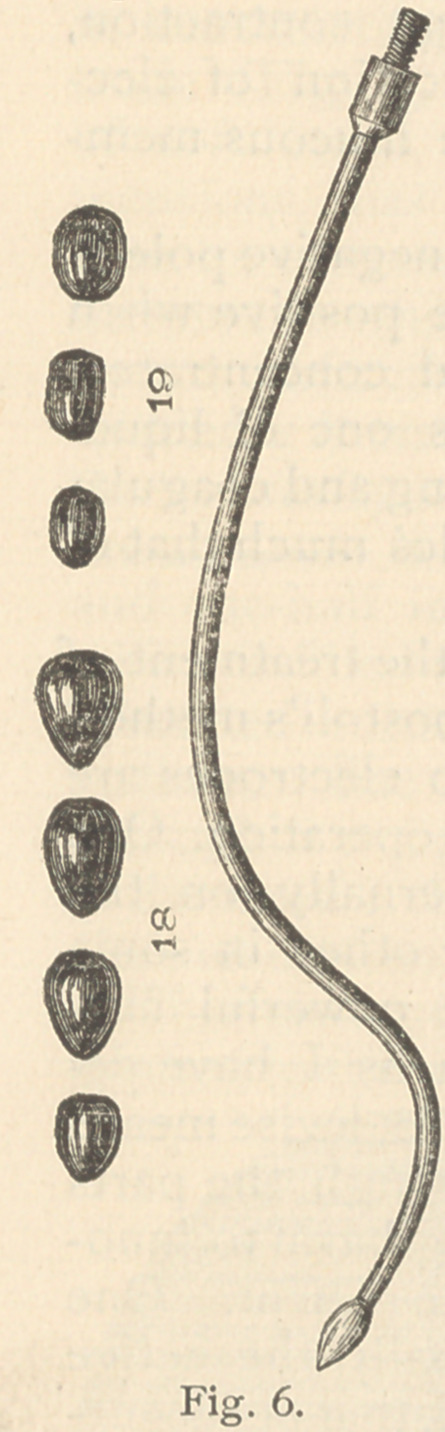


**Fig. 7. f8:**
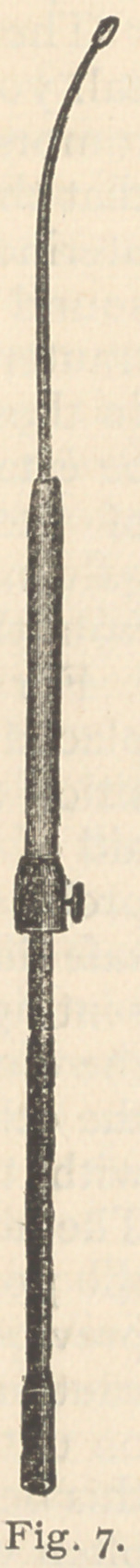


**Fig. 8. f9:**
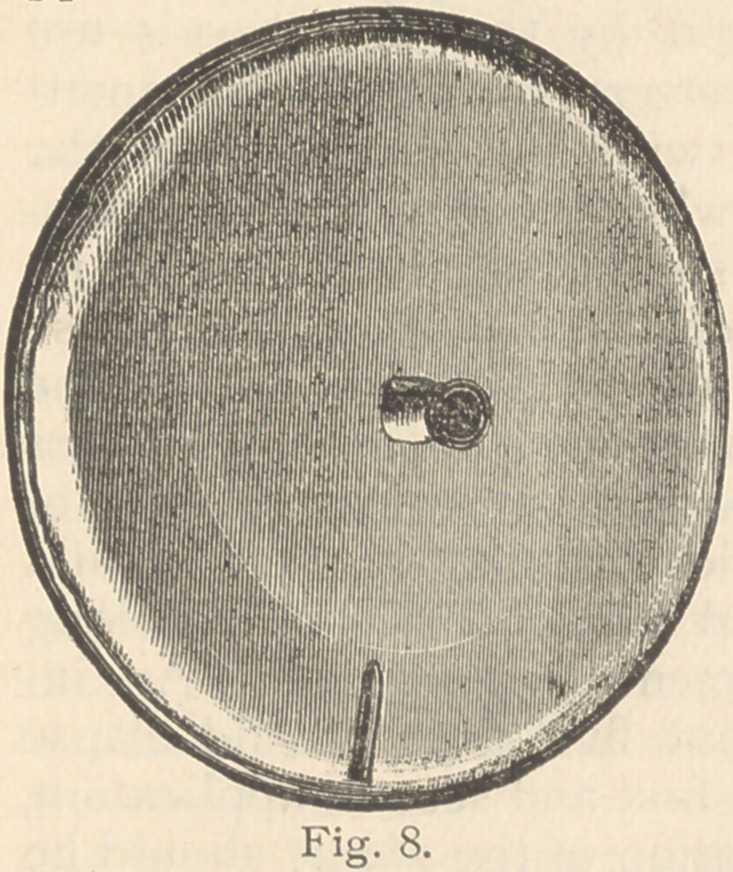


**Fig. 9. f10:**
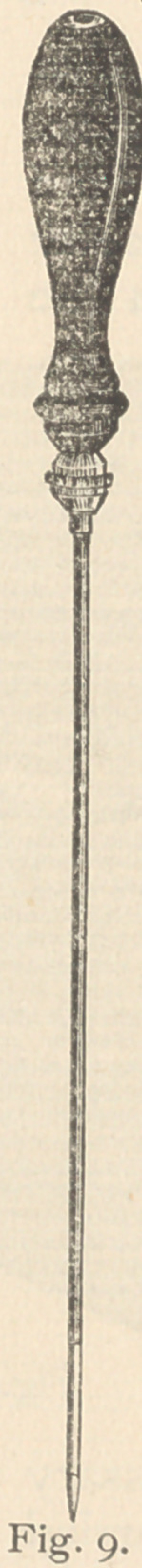


**Fig. 10. f11:**
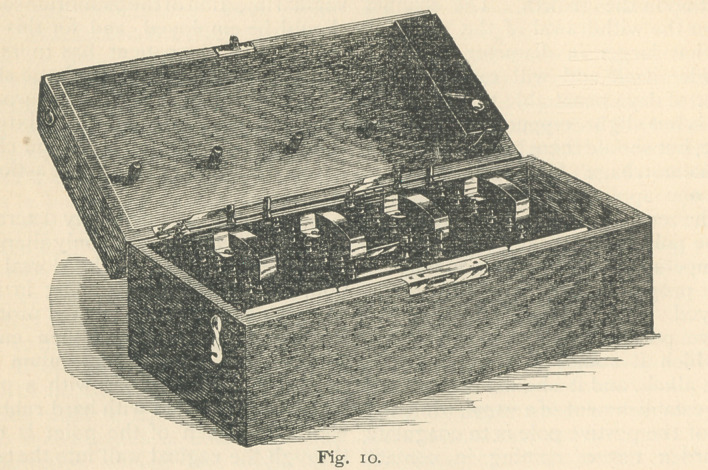


**Fig. 11. f12:**